# Return to Sport After Shoulder Arthroplasty: A Scoping Review

**DOI:** 10.7759/cureus.100696

**Published:** 2026-01-03

**Authors:** Daniel P McGurren, Corey Peacock

**Affiliations:** 1 Physical Therapy, Nova Southeastern University Dr. Kiran C. Patel College of Osteopathic Medicine, Fort Lauderdale, USA; 2 Health and Human Performance, Nova Southeastern University Dr. Kiran C. Patel College of Osteopathic Medicine, Fort Lauderdale, USA

**Keywords:** orthopaedics and sports physical therapy, return to sport, return to sport testing, reverse total shoulder arthroplasty, total shoulder arthroplasty

## Abstract

This scoping review synthesized existing literature on return-to-sport (RTS) protocols and testing criteria following total shoulder arthroplasty (TSA) to identify gaps in evidence-based practice and inform future research priorities for clinicians. A comprehensive literature search was performed using PubMed and Google Scholar from January 2015 to September 2025, following Preferred Reporting Items for Systematic Reviews and Meta-Analyses extension for Scoping Reviews (PRISMA-ScR) guidelines. Inclusion criteria comprised studies involving adults who underwent TSA and reported RTS protocols or testing criteria. Twenty-three studies published between January 2015 and September 2025 met the inclusion criteria. RTS rates were high (85.1% overall, 72.3% at equivalent or improved levels), with anatomical TSA (aTSA) demonstrating superior rates (92.6%) compared to hemiarthroplasty (HSA; 71.1%) or reverse TSA (rTSA; 74.9%). Average return time ranged from four to nine months across sports. However, objective testing criteria for clearance were notably absent. Most studies relied on time-based restrictions and subjective physician assessment without standardized measurement protocols or operational definitions for range of motion (ROM) and strength requirements. Despite encouraging RTS rates, a significant gap exists between successful outcomes and available evidence-based clearance criteria. Current protocols rely on arbitrary time-based restrictions rather than objective functional measures. Critical deficiencies include the absence of standardized strength testing, sport-specific assessments, validated outcome measures, and long-term implant survival data.

## Introduction and background

Shoulder arthroplasty surgeries, including hemiarthroplasty (HSA), anatomic total shoulder arthroplasty (aTSA), and reverse total shoulder arthroplasty (rTSA), have become increasingly common for patients with varying end-stage pathologies. Total shoulder arthroplasties (TSAs) now represent the third most common joint replacement procedure, with an annual total of nearly 200,000 procedures per year [1]. As the population of the United States continues to age, osteoarthritis and TSA procedure volume are projected to increase [2].

The patient population undergoing shoulder arthroplasty has evolved significantly over the past two decades, with younger, more active individuals increasingly receiving these procedures due to post-traumatic arthritis, inflammatory conditions such as rheumatoid arthritis, and massive rotator cuff tears with cuff tear arthropathy [2]. For these patients, the ability to return to meaningful athletic activities significantly influences both patient satisfaction and perceived surgical success, representing a primary outcome measure that directly affects overall quality of life [2]. This demographic shift, combined with improved prosthetic designs and surgical techniques, promises greater longevity. Consequently, this has created both opportunity and responsibility for clinicians to develop evidence-based approaches to athletic return.

This shift towards younger, more active patients makes return to sport (RTS) after TSA an increasingly important outcome measure in contemporary orthopedic practice. The growing body of research suggests that the majority of patients who undergo shoulder arthroplasty can successfully return to recreational and competitive athletic activities, with return rates varying by implant type and sport. Common sports studied include swimming, golf, fitness activities, and cycling, with return rates influenced by the biomechanical demands and loading patterns of each activity. Despite these encouraging outcomes, questions remain regarding the optimal timing, criteria, and protocols for safe return to athletic participation.

Rehabilitation guidelines exist for shoulder arthroplasty, and protocols typically recommend phased approaches to recovery, progressing from initial protection and passive motion through active strengthening before eventual sport participation, but they lack validated objective criteria, including specific range of motion (ROM) thresholds, strength benchmarks using dynamometry or limb symmetry indices, and functional performance measures to determine sport readiness. These protocols commonly suggest restricting high-demand upper extremity sports for several months postoperatively to allow for tissue healing and functional restoration. Despite the existence of these general frameworks, significant gaps persist regarding sport-specific protocols accounting for the distinct biomechanical demands of different activities, and long-term implant survival data in athletic populations remain limited. Therefore, identifying objective measures to guide this process remains a pressing need in contemporary orthopedic care.

Given the growing volume of TSA procedures and the increasing emphasis on maintaining active lifestyles across all age groups, establishing evidence-based RTS protocols has become increasingly important. This scoping review aims to systematically map the existing literature on RTS protocols and objective testing criteria following TSA, identify critical gaps in evidence-based practice, and inform future research priorities to guide clinicians in safely returning athletes and active individuals to sport following shoulder arthroplasty procedures. Specifically, this review addresses the following questions: (1) What evidence exists regarding RTS protocols and objective testing criteria for patients following TSA? (2) What specific clearance criteria, including ROM thresholds, strength measures, and functional assessments, are currently employed to determine sport readiness? (3) How do RTS outcomes and timeframes vary by implant type and sport activity?

## Review

**Figure 1:**
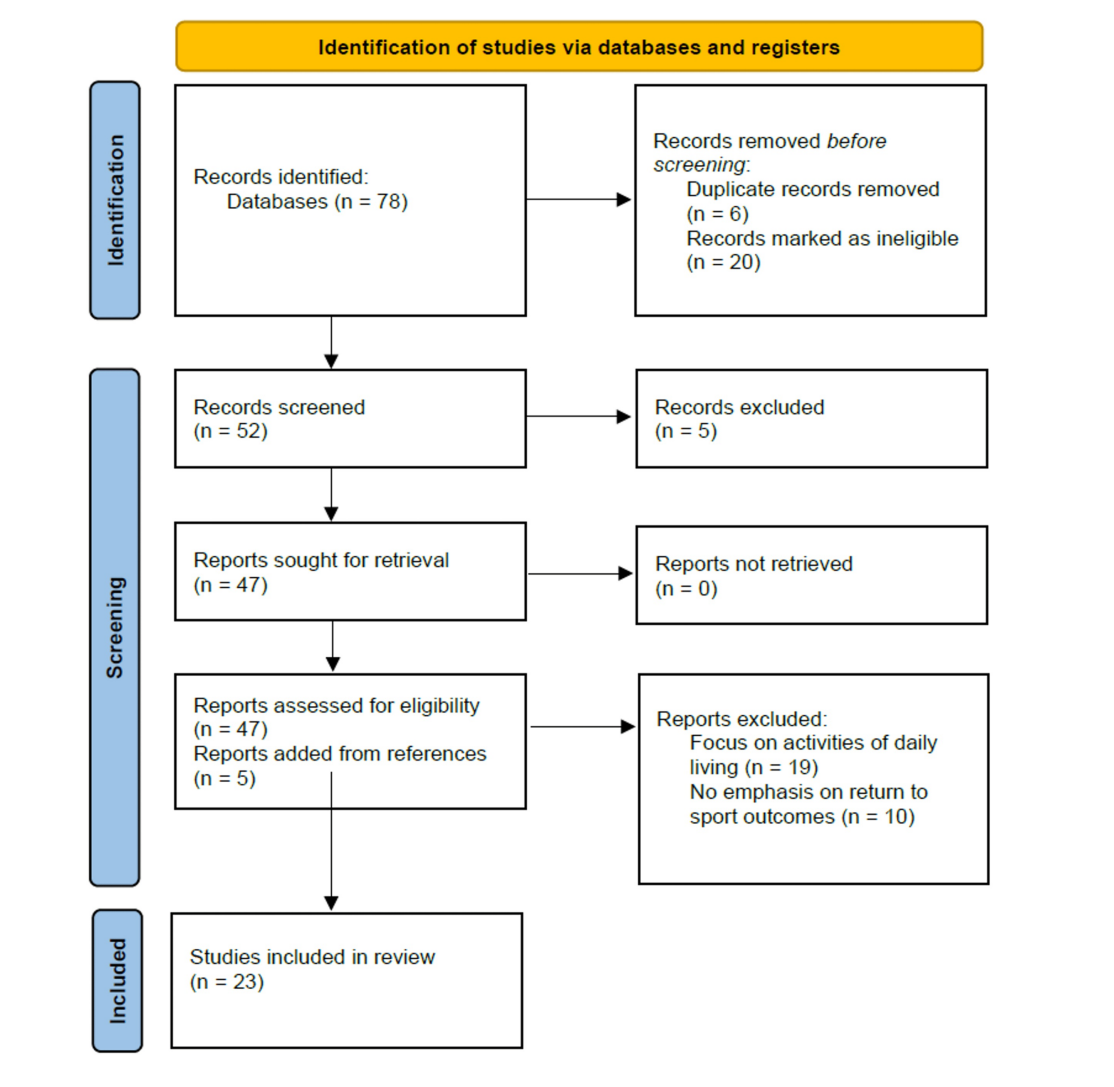
PRISMA-ScR 2020 Flow Diagram PRISMA-ScR = Preferred Reporting Items for Systematic Reviews and Meta-Analyses – Extension for Scoping Reviews

This literature review was conducted in accordance with the Preferred Reporting Items for Systematic Reviews and Meta-Analyses Extension for Scoping Reviews (PRISMA-ScR) guidelines and followed the methodological framework outlined by Arksey and O'Malley [3]. The review methodology was selected to map the existing literature on RTS protocols and testing criteria following TSA, identify research gaps, and synthesize diverse study designs and outcome measures within this emerging field. A summary of the PRISMA methodology is presented in Figure 1 [4].

A comprehensive literature search was performed using multiple electronic databases, including PubMed, from January 2015 through September 2025. The search strategy employed a combination of Medical Subject Headings (MeSH) terms and keywords. Primary search terms included the following: shoulder arthroplasty terms: "total shoulder arthroplasty," "reverse total shoulder arthroplasty," "anatomic total shoulder arthroplasty," "hemiarthroplasty," "shoulder replacement," "shoulder prosthesis"; RTS terms: "return to sport," "return to play," "athletic participation," "sports participation," "recreational activities"; protocol and testing terms: "rehabilitation protocol," "return to sport protocol," "functional testing," "clearance criteria," "sport-specific testing," "performance testing." Reference lists of included studies and relevant systematic reviews were hand-searched to identify additional eligible studies.

Inclusion criteria comprised (1) studies involving human participants aged ≥18 years who underwent TSA (including aTSA, rTSA, or HSA); (2) studies reporting on RTS outcomes, protocols, rehabilitation guidelines, or testing criteria; and (3) studies published in the English language. Exclusion criteria included studies focusing exclusively on activities of daily living without sport-specific outcomes, conference abstracts, editorials, opinion pieces, and case reports with fewer than five participants; studies not available in the English language; and studies focusing solely on surgical technique without rehabilitation or RTS outcomes. Data extraction was performed systematically, capturing study characteristics (design, sample size, publication year), patient demographics, implant types, RTS rates and timeframes, rehabilitation protocols, objective clearance criteria, and sport-specific outcomes. Given the heterogeneity in study designs and outcome measures, a narrative synthesis approach was employed to summarize findings.

Study selection and characteristics

A total of 78 articles were identified through database searches using PubMed and Google Scholar. After screening titles and abstracts, 26 articles were marked as ineligible due to language limitations and duplicates. After screening abstracts against the inclusion and exclusion criteria, 5 more articles were excluded. After a deeper review of these articles, 19 were excluded for focusing on return to activities of daily living, and 10 had no significant emphasis on RTS. Hand-searching of reference lists from the remaining eligible articles yielded five additional studies. In total, 23 publications met all inclusion criteria and were included in this scoping review.

The included studies comprised systematic reviews, meta-analyses, and cohort studies published between January 2015 and September 2025. Study populations ranged from recreational athletes to competitive participants across various age groups. The most commonly studied sports included golf, swimming, cycling, and fitness activities such as lightweight training. Implant types examined included aTSA, rTSA, and HSA, with some studies comparing outcomes across implant types. A detailed summary of these findings is provided in Table 1.

**Table 1:** Key Studies and Findings in RTS After Shoulder Arthroplasty RTS = Return to Sport; aTSA = Anatomic Total Shoulder Arthroplasty; rTSA = Reverse Total Shoulder Arthroplasty; HSA = Hemiarthroplasty; ROM = Range of Motion; ASES = American Shoulder and Elbow Surgeons; ASSET = American Society of Shoulder and Elbow Therapists; APTA = American Physical Therapy Association; OA = Osteoarthritis

Study (Year)	Study Type	Implant Type	Key Findings	RTS Timeframe	Objective Criteria	Sport-Specific Data
Shimada et al. (2023) [5]	Cohort study	aTSA, rTSA	aTSA: 93% overall RTS, 70% complete return. rTSA: 83% overall RTS, 30% complete return	>2 years mean follow-up	Not specified	High-load activities: aTSA 70%, rTSA 30% complete return
Papalia et al. (2020) [6]	Systematic review & meta-analysis	aTSA, rTSA	Overall RTS: 82%. aTSA: 90%, rTSA: 77%. Return to same/higher level: 75%	Not specified	Not specified	Swimming: 84%, Fitness: 77%, Golf: 77%, Tennis: 69%
Salem et al. (2021) [7]	Systematic review	TSA, HSA	TSA golf RTS: 89-100%. HSA golf RTS: 54%	TSA: 5.1-8.4 months. HSA: 6.5 months	Not specified	Golf-specific study
Cvetanovich et al. (2020) [8]	Cohort study	aTSA	75% returned to sport. 50% returned to the same/higher level	Average 9.1 months	Not specified	Younger, active patients studied
Mousad et al. (2025) [9]	Cohort study	TSA	Swimming RTS: 82%. Most resumed primary stroke with improved ability/enjoyment	Not specified	Not specified	Swimming-specific study
Davey et al. (2021) [10]	Systematic review	rTSA	Overall RTS: 79.1%. Return to same level: 71.4%	3-6 months	Not specified	Golf: 66.7%, Swimming: 74.3%, Tennis: 50%, Jogging: 94.4%, Cycling: 69.7%
Geyer et al. (2022) [11]	Comparative study	rTSA	RTS: 78%. Athletes demonstrated superior clinical outcomes vs non-athletes	Average 5.3 months	Not specified	Not specified
Garcia et al. (2016) [12]	Cohort study	HSA	RTS to at least one sport: 67.2%	Average 6.5 months (range 4-7)	Not specified	Fitness: 69%, Swimming: 65%, Running: 64%
Liu et al. (2018) [13]	Systematic review & meta-analysis	aTSA, rTSA, HSA	Overall RTS: 85.1%; Return to equivalent/improved level: 72.3%. aTSA: 92.6%, rTSA: 74.9%, HSA: 71.1%	Within 6 months	Not specified	Swimming, golf, fitness, and cycling studied
Kennedy et al. (2020) [14]	Consensus statement (ASES/ASSET)	aTSA	Three-phase protocol: Phase 1 (weeks 1-6): passive ROM, subscapularis protection. Phase 2 (weeks 6-12): active ROM progression. Phase 3 (12+ weeks): resistance, sport initiation	Advanced sports: 4 months. Full return: 6 months	Pain-free active ROM = passive ROM, scapulohumeral rhythm restoration, strength testing (deltoid, rotator cuff, scapular stabilizers), sport-specific functional analysis. NO operational definitions	High-demand sports not before six months. Impact loading discouraged
Polio & Brolin (2023) [15]	Review article	aTSA	Postoperative rehabilitation protocol discussion	Advanced sports: 4 months. Full return: 6 months	Not specified	Not specified
Cronin et al. (2021) [16]	Cohort study	TSA	Patient-reported outcome measures responsiveness analysis	Not specified	ASES score, Single Assessment Numeric Evaluation, Simple Shoulder Test for readiness assessment. Radiographic confirmation of implant stability	Not specified
Beleckas et al. (2025) [17]	Cohort study	aTSA, rTSA	Evaluation of "new normal" after shoulder arthroplasty	Clearance at/after 6 months	No evidence of subscapularis dysfunction or implant-related issues required	Not specified
Michener et al. (2023) [18]	Clinical practice guideline (APTA)	TSA (glenohumeral OA)	Physical therapy management guidelines for glenohumeral joint osteoarthritis	Clearance at/after 6 months	Not specified	Not specified
Herring et al. (2024) [19]	Consensus statement	rTSA	Team physician consensus on return to sport/play - 2023 update	After month 6	Objective clearance criteria mentioned but not operationally defined	Non-contact sports permitted as tolerated in months 3-6
Lee et al. (2021) [20]	Cohort study	rTSA	Accelerated rehabilitation following rTSA studied	Not specified	Not specified	Not specified
Lu et al. (2022) [21]	Systematic review	rTSA	Clinical outcomes of physiotherapy after rTSA analyzed	Not specified	Not specified	Not specified
Garcia et al. (2016) [22]	Matched comparison	HSA, TSA	Hemiarthroplasty vs TSA for shoulder osteoarthritis comparison	3 months most common clearance	Clinical and functional assessment, patient-reported outcomes, and absence of pain/instability	Return to sports comparison
Liu et al. (2016) [23]	Comparative analysis	HSA, rTSA	Sports after shoulder arthroplasty: comparative analysis	4-9 months	Not specified	Comparative sport participation data
Bülhoff et al. (2018) [24]	Cohort study	Humeral head resurfacing	Partial return at 3 months. Full return 7-12 months for majority	Partial: 3 months. Full: 7-12 months	Not specified	Progressive loading approach emphasized
Aim et al. (2018) [25]	Systematic review & meta-analysis	TSA	Return to sport in recreational athletes analyzed	Golf: 4.5-8.4 months. Swimming: 6.7 months. Cycling: 6.5 months	Not specified	Sport-specific return times with biomechanical consideration
Mannava et al. (2018) [26]	Cohort study	TSA	Return to recreational sporting activities studied	4-6 months typical clearance	"Full ROM and strength" required - NO operational definitions provided (no degree measurements, percentages, dynamometry values, or limb symmetry indices)	Recreational sporting activities
Kim et al. (2023) [27]	Cohort study	rTSA	Successful RTS patients had significantly greater forward flexion than non-RTS patients	Not specified	Forward flexion: Successful RTS 141.2° ± 10.9° vs Non-RTS 131.0° ± 18.2° (p=0.03). ~140° threshold suggested as potential criterion	Not specified

RTS rates and outcomes

RTS rates and timeframes after shoulder arthroplasty vary significantly by procedure type, with aTSA consistently demonstrating the highest rates, followed by rTSA, and then HSA. In the general athletic population, the literature provides robust quantitative data to guide preoperative counseling and expectation management.

For aTSA, the overall RTS rate ranges from 75% to 93%, with most patients returning to sport within four to six months postoperatively. Shimada et al. reported a 93% overall RTS rate and a 70% complete return rate at a mean follow-up exceeding two years [5]. Papalia et al. found a pooled RTS rate of 90% in elderly patients, with 75% returning to the same or higher level of sport [6]. For specific sports, Salem et al. reported RTS rates for golf of 89% to 100% after TSA, with mean return times of 5.1 to 8.4 months [7]. Cvetanovich et al. found that 75% of younger, active patients returned to sport at an average of 9.1 months, with 50% achieving the same or higher level of activity [8]. Swimming-specific data from Mousad et al. showed an 82% RTS rate after TSA, with most patients resuming their primary stroke and reporting improved ability and enjoyment [9].

rTSA yields RTS rates of 75% to 83%, with return typically occurring within three to six months. Davey et al. reported an overall RTS rate of 79.1% after rTSA, with 71.4% returning to the same level of sporting activity [10]. Sport-specific rates include 66.7% for golfers, 74.3% for swimmers, 50% for tennis players, 94.4% for joggers, and 69.7% for cyclists [9]. Shimada et al. found an 83% overall RTS rate for rTSA, but only 30% achieved a complete return [5]. Geyer et al. reported a 78% RTS rate at an average of 5.3 months, with athletic patients demonstrating superior clinical outcomes compared to non-athletes [11].

HSA is associated with the lowest RTS rates, ranging from 54% to 67%, with most patients returning within four to seven months. Salem et al. reported a 54% RTS rate for golf after HSA, with a mean return time of 6.5 months [7]. Garcia et al. found a 67.2% rate of RTS at least one sport at an average of 6.5 months, with higher rates for fitness sports (69%), swimming (65%), and running (64%) [12]. Liu et al. reported a 71.1% RTS rate for HSA, compared to 92.6% for TSA and 74.9% for rTSA, with most patients returning within six months [13].

In summary, aTSA offers the highest likelihood of returning to sport (75%-93%) with the best chance of returning to pre-injury level, while rTSA provides reliable but slightly lower rates (75%-83%), and HSA demonstrates the most limited athletic recovery (54%-67%). Regardless of procedure type, most patients who successfully RTS do so within six months, though individual sport demands and patient-specific factors significantly influence both the rate and level of return.

Rehabilitation protocols and objective clearance criteria

Throughout the literature, rehabilitation protocols and objective clearance criteria for RTS after shoulder arthroplasty are best defined for aTSA, with less standardization for rTSA and HSA. The American Society of Shoulder and Elbow Surgeons (ASES) and the American Society of Shoulder and Elbow Therapists (ASSET) have published detailed consensus guidelines for aTSA, while protocols for rTSA and HSA are often extrapolated from aTSA or based on expert opinion [14].

*Anatomic *​​​*TSA*

Rehabilitation after an aTSA follows a three-phase, consensus-based protocol. Phase 1 (postoperative day 1 to week 4-6) involves sling immobilization, passive ROM, and protection of the subscapularis and joint capsule. Precautions include avoiding hands behind the back and external rotation beyond 30°, with no active shoulder elevation. Phase 2 (week 4-6 to week 12) discontinues the sling and gradually restores active ROM to match passive ROM, allowing external rotation at 90° abduction up to 60° and gentle internal rotation behind the back. Weight-bearing is permitted for assistive device use, but closed-chain exercises are avoided. Phase 3 (week 12+) introduces resistance exercises with low loading and high repetition, restricting weight training to below shoulder level and anterior to the frontal plane. Closed-chain activities such as planks and yoga are permitted, and a gradual RTS is initiated. Advanced upper extremity sports (e.g., golf, tennis) may begin at four months, with full return to play at six months to allow for mature subscapularis healing [14,15].

Objective clearance for RTS is based on pain-free active ROM equal to passive ROM in all planes, restoration of scapulohumeral rhythm, strength testing of the deltoid, rotator cuff, and scapular stabilizers, and sport-specific functional analysis. Thirteen patient-reported outcome measures (PROMs), such as the ASES score, Single Assessment Numeric Evaluation, and Simple Shoulder Test, are used to assess readiness, with radiographic assessment confirming implant stability [16]. Clearance for full RTS is typically granted at or after six months, provided these criteria are met, and there is no evidence of subscapularis dysfunction or implant-related issues [17,18].

Reverse TSA

Rehabilitation after rTSA is less standardized but generally follows a staged approach. The immediate postoperative phase (weeks 0-4) focuses on sling immobilization, passive ROM, and protection of the surgical repair. Early scapular mobility and elbow/wrist/hand exercises are encouraged. The early rehabilitation phase (weeks 4-8) introduces active-assisted and active ROM, with isometric strengthening of the deltoid and periscapular muscles. The intermediate phase (weeks 8-12) progresses to strengthening exercises and sport-specific conditioning. The advanced phase (months 3-6) emphasizes full active ROM, advanced strengthening, and sport-specific drills, with non-contact sport activities permitted as tolerated. The return to sport phase (after month 6) involves gradual reintroduction to full sport participation, contingent on meeting objective clearance criteria [19-21].

Objective clearance for RTS after rTSA includes assessment of pain, ROM, strength, and validated outcome measures such as the Constant score, ASES score, and Simple Shoulder Test [5]. Radiographic monitoring for implant-related complications is recommended, though incomplete radiolucency around the humeral component, while more common in athletic patients, does not correlate with higher rates of loosening or revision [10,11]. Psychosocial readiness is also assessed, with the physician responsible for confirming satisfactory status in all domains before clearing an athlete for RTS.

Hemiarthroplasty

Rehabilitation protocols for HSA are the least well-defined in the literature. Most protocols mirror those for anatomic TSA, with a staged approach focusing on pain control, restoration of passive and active ROM, strengthening, and sport-specific training. The typical timeframe for RTS is four to seven months, with clearance based on clinical and functional assessment, patient-reported outcomes, and absence of pain or instability [7,12-14,22]. There is a lack of standardized, validated objective clearance tests specific to HSA, and most studies rely on clinical judgment and patient-reported improvement.

Sport clearance guidelines

Time-Based Clearance

Multiple studies reported on temporal parameters for return to various sporting activities, with considerable variation observed across both sports and studies. Overall, most investigations reported average return times ranging from four to nine months postoperatively, with standard recommendations maintaining restrictions until at least six months [13,22-24].

Bülhoff et al. provided the most detailed timeline for RTS progression following humeral head resurfacing [24]. Their cohort study reported that partial RTS typically occurred around three months postoperatively, with the majority of athletes achieving full return within seven to 12 months. This graduated approach to sport resumption reflects clinical practice patterns that emphasize progressive loading and activity advancement rather than immediate return to preoperative participation levels.

Sport-specific return times varied considerably based on the biomechanical demands and intensity of the activity. For golf, reported average return times ranged from 4.5 to 8.4 months across multiple studies [6,13,25]. Swimming demonstrated a more consistent average return time of 6.7 months, while cycling averaged 6.5 months postoperatively [6,13,25]. The substantial range in golf return times (nearly four months) may reflect differences in study populations, implant types, or varying definitions of what constitutes successful RTS.

Objective Testing Criteria

There is a significant lack of evidence for objective data for RTS clearance. Mannava et al. reported that subjects in their cohort were cleared for full recreational sporting activities after achieving full ROM and strength [26]. However, the study provided no operational definitions for the "full" ROM (e.g., specific degree measurements, percentage of contralateral limb, or comparison to normative data) or strength (e.g., manual muscle testing grades, dynamometry values, or limb symmetry indices). Furthermore, clearance remained contingent on time, with four to six months identified as the typical timeframe for clearance to sporting activities, suggesting that temporal criteria superseded or complemented functional measures.

Kim et al. provided the only study identifying specific quantitative measurements associated with successful RTS outcomes [27]. In their analysis of patients undergoing rTSA, those who successfully returned to sport demonstrated significantly greater forward flexion compared to those who did not return (141.2° ± 10.9° versus 131.0° ± 18.2°, p = 0.03). While this study did not utilize this measurement as a prospective clearance criterion, the statistically significant difference suggests that a forward flexion threshold of approximately 140° may differentiate successful from unsuccessful RTS candidates and could serve as a potential objective criterion in future protocols.

The remaining studies reported that clearance was contingent upon physician examination and assessment; however, none specified what components comprised these examinations, what objective tests were performed, or what standards were used to determine readiness. This lack of specificity reflects the current state of practice, where RTS decisions appear to rely primarily on subjective clinical judgment rather than validated, objective criteria.

Sport-Specific Considerations

RTS rates for high-demand or contact sports are consistently lower than for low-demand, non-contact activities. Shimada et al. classified sports and physical work as low-, medium-, or high-load activities and found that, following anatomic TSA, the complete return rate for high-load activities was 70%, compared to 30% for rTSA [5]. Tennis, as a representative high-demand overhead sport, has RTS rates of 50% after rTSA and 69% after TSA or rTSA in elderly patients [6]. The ASES and the ASSET recommend that a full return to high-demand or contact sports not occur prior to six months postoperatively, with impact loading activities such as sledgehammer use, wood chopping, and bench pressing discouraged after anatomic TSA [14].

None of the included studies reported the use of sport-specific return to play protocols that accounted for the distinct biomechanical demands of different athletic activities. While several studies stratified outcomes by sport type (e.g., golf, swimming, cycling), the actual clearance criteria and rehabilitation progressions did not differ based on the sport to which patients intended to return [6,13,22,23,26,27]. This one-size-fits-all approach fails to address the vastly different physical requirements and risk profiles associated with various sports, from low-impact activities such as cycling to high-demand overhead sports such as tennis or swimming.

Discussion

This scoping review examined the current evidence regarding RTS protocols and testing criteria following shoulder arthroplasty across 23 studies published between January 2015 and September 2025. The findings reveal a striking paradox: while RTS rates are highly encouraging across all implant types, the evidence base for objective clearance criteria is remarkably thin, with minimal studies providing specific quantitative thresholds.

RTS Success Despite Evidence Gaps

The quantitative outcomes demonstrate considerable success in returning patients to athletic activity. Among the three primary implant types examined, aTSA achieved the highest return rates (75%-93%), with most patients resuming activities within four to six months. rTSA demonstrated slightly lower but still robust rates (75%-83%) with similar timeframes (three to six months), while hemiarthroplasty yielded the lowest return rates (54%-67%) with return occurring at four to seven months [5,7,10-13,28] These data align with the findings of Liu et al., who reported 85.1% overall RTS, with 72.3% returning to equivalent or improved levels of play, and Papalia et al., who found 82% overall return rates [6,13].

Sport-specific return rates varied considerably by activity type. Swimming demonstrated the highest return rate at 84%, followed by fitness activities and golf at 77% each, while tennis showed lower rates at 69% [6]. These variations likely reflect the distinct biomechanical demands and loading patterns inherent to each sport. However, the critical limitation is that while these outcome data are robust, the criteria used to clear patients for return remain largely undefined.

Absence of Standardized Objective Criteria

The most significant finding of this review is the near-complete absence of objective, quantifiable testing criteria for RTS clearance. Of the included studies, only Kim et al. provided specific quantitative measurements, identifying that patients who successfully returned to sport after rTSA demonstrated significantly greater forward flexion compared to those who did not (141.2° ± 10.9° versus 131.0° ± 18.2°, p = 0.03) [27]. While this suggests a potential threshold of approximately 140° of forward flexion may differentiate successful from unsuccessful return candidates, this isolated finding was observational rather than a prospectively applied clearance criterion.

Mannava et al. referenced requirements for "full range of motion and strength" before clearance but provided no operational definitions for these terms, no specific degree measurements, percentages of contralateral limb function, normative data comparisons, manual muscle testing grades, dynamometry values, or limb symmetry indices [26] The remaining studies offered even less specificity, stating only that clearance was based on "physician assessment" or "examination" without describing what components comprised these evaluations or what standards determined readiness. This lack of specificity means that current practice appears to rely primarily on subjective clinical judgment rather than validated, reproducible objective measures.

Time-Based Versus Function-Based Approach

Current practice predominantly employs time-based restrictions, with most studies reporting average return timeframes of four to nine months postoperatively. The ASSET and ASES consensus guidelines exemplify this approach, recommending advanced upper extremity sports at four months with full return after six months, contingent primarily on subscapularis healing rather than functional achievement [14]. Bülhoff et al.'s findings of partial return at three months and full return at seven to 12 months, along with Garcia et al.'s identification of three months as the most common clearance timepoint, demonstrate considerable practice variation [22,24].

This time-based approach fails to account for substantial individual variability in healing rates, preoperative functional status, surgical complexity, rehabilitation adherence, patient age, and other factors that significantly influence recovery trajectories. While some clinicians may be individualizing return timelines based on clinical judgment, the absence of validated objective criteria means such individualization lacks the standardization necessary to optimize outcomes while minimizing risks of re-injury or prosthetic complications.

Rehabilitation Protocol Standardization

The degree of protocol standardization varied markedly by implant type. aTSA benefits from the most comprehensive guidance, with detailed three-phase consensus protocols from ASES and ASSET delineating specific timeframes for sling discontinuation, range of motion progression, and strengthening advancement [14,15]. Objective clearance criteria for aTSA reference "pain-free active ROM equal to passive ROM in all planes," "restoration of scapulohumeral rhythm," and "strength testing of deltoid, rotator cuff, and scapular stabilizers," yet operational definitions for these assessments remain absent [14].

rTSA protocols are less standardized, generally following staged approaches but lacking the detailed consensus that exists for aTSA [19-21]. HSA protocols are the least defined, with most studies defaulting to aTSA frameworks without addressing the distinct biomechanical and healing considerations inherent to this procedure [7,12-14,22]. This graduated level of protocol development reflects both the relative procedural volumes and the research attention each implant type has received, highlighting the need for uniformity across procedure types.

Sport-Specific Considerations

Despite clear evidence that return rates vary by sport and activity intensity, none of the included studies described sport-specific return protocols that accounted for the distinct biomechanical demands of different athletic activities. Shimada et al. demonstrated that high-load activities achieved only 70% complete return rates after aTSA compared to 30% after rTSA, and tennis players showed reduced return rates (50% after rTSA, 69% overall) compared to lower-demand sports like swimming (84%) or cycling [5,6,10]. Yet clearance criteria and rehabilitation progressions remained uniform regardless of the sport to which patients intended to return.

This one-size-fits-all approach inadequately addresses the vastly different physical requirements between sports. Golf demands repetitive high-velocity rotation, potentially stressing subscapularis repairs; swimming requires sustained overhead endurance and neuromuscular control in challenging positions, while contact sports present distinct concerns regarding prosthetic stability and periprosthetic fracture risk [29, 30]. The absence of sport-specific protocols represents a missed opportunity to optimize both safety and performance outcomes.

Comparison to Lower Extremity Arthroplasty Standards

The gap between shoulder and lower extremity arthroplasty RTS protocols is striking. Hip and knee arthroplasty rehabilitation routinely employs validated strength measures, including handheld dynamometry and isokinetic testing with specific limb symmetry indices (typically ≥85%-90% compared to contralateral limb or normative data), functional performance measures such as hop tests and timed up-and-go assessments, and gait analysis to inform clearance decisions [31-33]. In contrast, shoulder arthroplasty appears to lack comparable assessment tools despite the complex biomechanical demands placed on the shoulder during athletic activities.

Limitations

This review has several limitations that warrant consideration. The search was restricted to English-language publications from January 2015 through September 2025, potentially excluding relevant international perspectives and earlier foundational work. The limited number of included studies reflects the nascent state of this research area but constrains the generalizability of conclusions. The heterogeneity in study designs, outcome measures, implant types, and patient populations precluded meta-analysis, limiting the ability to generate pooled effect estimates or definitive quantitative recommendations. Additionally, the included studies varied in their definitions of "RTS," with some considering any athletic participation as a successful return, while others required a return to equivalent or higher levels of performance.

Future Directions and Clinical Implications

Overall, the disconnect between successful return outcomes and available evidence-based clearance criteria demands urgent attention from the shoulder arthroplasty community. Future research priorities should include: (1) prospective studies establishing evidence-based thresholds for ROM, strength (including rotator cuff and scapular stabilizers), and functional performance that predict successful RTS; (2) development and validation of sport-specific functional testing batteries designed for the TSA population, with psychometric evaluation to establish reliability, validity, and normative data; (3) investigation of objective criteria that move beyond arbitrary time-based restrictions to capture the multidimensional nature of sport readiness; (4) long-term studies examining the impact of various RTS timelines and criteria on implant survival and revision rates; and (5) comparative effectiveness research evaluating different protocol approaches to identify optimal strategies.

As the population undergoing shoulder arthroplasty continues to trend younger and more active, the need for sophisticated, evidence-based RTS decision-making intensifies. While current return rates approaching 85% overall are encouraging, the field cannot continue to rely on subjective physician assessment and arbitrary timeframes without specific operational definitions. The establishment of validated, objective clearance criteria would not only enhance patient safety and optimize outcomes but also provide the evidence base necessary to counsel patients appropriately regarding expectations and risk. Until such criteria are developed and validated, clinicians must acknowledge these limitations while striving to incorporate the best available evidence, although limited, into individualized RTS decisions that honor both the desires of active patients and the responsibility to protect them from avoidable complications, ultimately bridging the gap between clinical success and athletic performance [34]

## Conclusions

This scoping review reveals a critical gap in the management of RTS following TSA. While RTS rates are highly encouraging, the evidence base for objective clearance criteria remains remarkably underdeveloped. Current practice relies predominantly on arbitrary time-based restrictions and subjective physician assessment without standardized operational definitions for ROM, strength thresholds, or functional performance measures. This absence of validated, objective criteria stands in stark contrast to the well-established protocols used in lower extremity arthroplasty and represents a significant opportunity for advancement in shoulder arthroplasty care. As the patient population undergoing these procedures continues to trend younger and more active, the development of evidence-based, sport-specific clearance protocols incorporating objective strength testing, functional performance measures, and validated outcome assessments is essential. Future research should prioritize establishing these standards to optimize both patient safety and athletic performance outcomes, ensuring that clinical decision-making moves beyond subjective judgment to become grounded in reproducible, validated criteria that can guide individualized RTS decisions for this increasingly active patient population.
